# Plasticity in the Macromolecular-Scale Causal Networks of Cell Migration

**DOI:** 10.1371/journal.pone.0090593

**Published:** 2014-02-28

**Authors:** John G. Lock, Mehrdad Jafari Mamaghani, Hamdah Shafqat-Abbasi, Xiaowei Gong, Joanna Tyrcha, Staffan Strömblad

**Affiliations:** 1 Center for Biosciences, Department of Biosciences and Nutrition, Karolinska Institutet, Huddinge, Sweden; 2 Division of Mathematical Statistics, Department of Mathematics, Stockholm University, Stockholm, Sweden; Seoul National University, Republic of Korea

## Abstract

Heterogeneous and dynamic single cell migration behaviours arise from a complex multi-scale signalling network comprising both molecular components and macromolecular modules, among which cell-matrix adhesions and F-actin directly mediate migration. To date, the global wiring architecture characterizing this network remains poorly defined. It is also unclear whether such a wiring pattern may be stable and generalizable to different conditions, or plastic and context dependent. Here, synchronous imaging-based quantification of migration system *organization*, represented by 87 morphological and dynamic macromolecular module features, and migration system *behaviour*, i.e., migration speed, facilitated Granger causality analysis. We thereby leveraged natural cellular heterogeneity to begin mapping the directionally specific causal wiring between *organizational* and *behavioural* features of the cell migration system. This represents an important advance on commonly used correlative analyses that do not resolve causal directionality. We identified *organizational* features such as adhesion stability and adhesion F-actin content that, as anticipated, causally influenced cell migration speed. Strikingly, we also found that cell speed can exert causal influence over *organizational* features, including cell shape and adhesion complex location, thus revealing causality in directions contradictory to previous expectations. Importantly, by comparing unperturbed and signalling-modulated cells, we provide proof-of-principle that causal interaction patterns are in fact plastic and context dependent, rather than stable and generalizable.

## Introduction

A key challenge in biology is to understand how information is coordinated globally within cells to generate and control complex cellular processes, such as cell migration. Succinctly, what is the wiring pattern of regulation that governs a particular cell behavior? Importantly, this raises a second fundamental question that we seek to address herein: is the wiring pattern for a particular process stable and generalizable, or, plastic and contextually dependent? The answer to this second question has important implications for our understanding of both complex biological processes and the design of the experimental strategies to address them.

Cell migration is a process of vital importance in numerous physiological and pathological processes including cancer cell metastasis [1]. Cell migration is indeed a highly complex cellular process, arising from a large, self-organizing molecular network to produce behaviors that are dynamic, heterogeneous and adaptable [2]. The dynamism of these behaviors suggests that underlying plasticity in the wiring of the cell migration system might be both more likely and more readily detectable than in relatively constrained cellular phenomena. Thus cell migration provides an appropriate framework within which to assess both the structure and potential plasticity of cellular wiring patterns.

**Figure 1:**
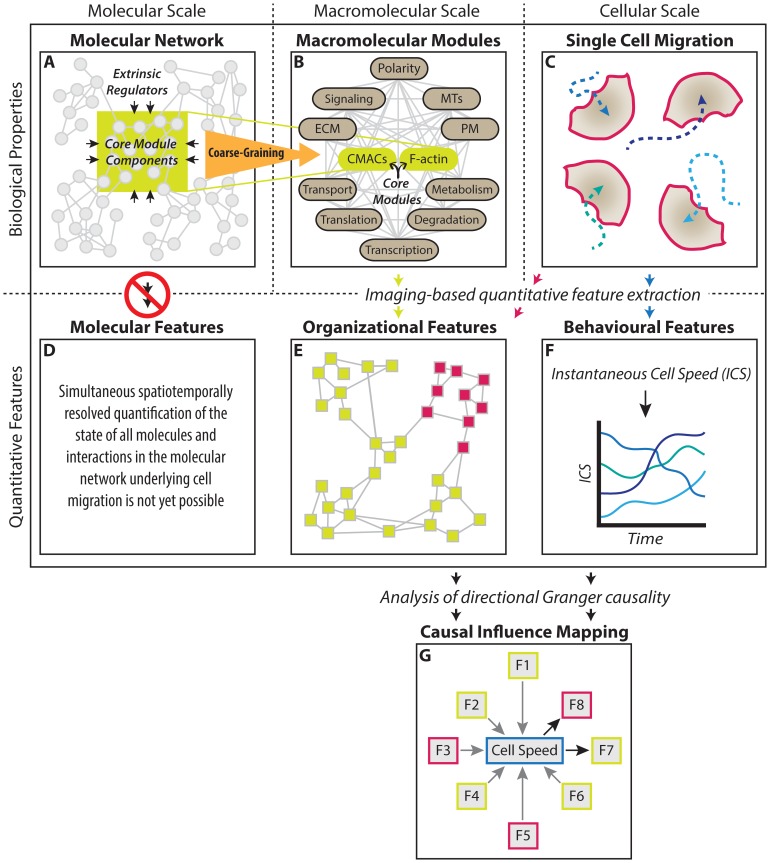
Rationale for a coarse-grained analysis of causal influence in the cell migration system. Cell migration emerges from biological properties encompassing multiple scales (A–C). At the molecular scale, thousands of distinct components and their interactions produce a complex and modular *molecular network* comprising the cell migration system (A). At the macromolecular scale, this network gives rise to a variety of functional *macromolecular modules* (B), which collectively produce *single cell migration* at the cellular scale (C). Unfortunately, it is not yet possible to synchronously record the state of the molecular network underlying cell migration with spatiotemporal resolution (D). Instead, we coarse-grain (orange arrow from A to B) this molecular complexity by focusing our analysis at the scale of macromolecular modules. Specifically, we focus on CMACs and the F-actin cytoskeleton (green ovals in B) because these *core modules* directly mediate the process of cell migration. Their observable features exemplify the state of both: i) their own molecular components (green box in A), and; ii) extrinsic sources of regulation distributed throughout the broader molecular network from which they integrate information (black arrows in A). This information is functionalized through adaptive changes in the *organization* of these core macromolecular modules and at the cellular-scale, leading to associated changes in migratory *behaviour*. Through imaging and quantitative analysis of individual migrating cells expressing EGFP-Paxillin and RubyRed-LifeAct (markers for CMAC and F-actin modules, respectively), we extract 88 quantitative features defining *organizational* (E) and *behavioural features* (F) of the cell migration system (*see Supporting Tables S1 and S2 for feature descriptions*). Briefly, *organizational* features include those describing core macromolecular module status (lime-green boxes in E, e.g. CMAC area, CMAC lifetime, RubyRed-LifeAct intensity within CMACs, etc) and cellular-scale morphological features (pink boxes in E, e.g. cell perimeter, cell compactness (roundness), number of CMACs per cell), while *behavioral* features addressed in this study relate exclusively to cell migration speed (F). Finally, Granger causality analysis enables *causal influence mapping* to define the nature and direction of causal information flow between pairs of these coarse-grained features of the cell migration system (G).

Cell migration is the product of interactions and interdependencies operating across molecular, macromolecular, and cellular scales (see Figure 1). As noted above, a huge diversity of components comprise the network underlying migration at the molecular scale (Figure 1 A) [3]–[8]. Such large-scale molecular networks tend to be arranged into hierarchically nested assemblages or modules [9], [10]. These macromolecular modules often represent functional units with distinct roles whose interactions ultimately produce single cell migration behaviors (Figure 1 B and C).

To understand how cell migration *behaviors* derive from molecular-, macromolecular- and cellular-scale *organization*, it is desirable to characterize all scales simultaneously and with sufficient spatiotemporal resolution to delineate functional relationships between features at any scale. Live cell fluorescence imaging provides the spatiotemporal resolution to track individual migrating cells while concurrently monitoring features of their molecular- and macromolecular-scale organization. Unfortunately, such imaging does not permit us to directly observe the complete state of molecular networks underlying cell migration (Figure 1 D), and as a consequence we cannot synchronously and globally observe how molecular signaling pathways are integrated. The canonical means to overcome such limitations on direct observation have been to assemble, piecewise, the functional contributions and relations of individual molecular components through perturbation-based epistasis analyses. Yet, despite facilitating great progress, reductionist perturbation-based approaches alone may be insufficient to provide a systems-level understanding, particularly of dynamic, heterogeneous processes with potentially emergent properties [11]–[14]. In particular, there are substantial risks of misattribution associated with the inference of molecular function based on targeted component perturbation [15]. Thus, there remain significant limitations on our ability to spatiotemporally resolve, either through direct observation or perturbation-based inference, how global information processing at the molecular scale gives rise to migratory behaviors.

Given the modularity in molecular network structure noted above, an alternative strategy is to analyze the state and interactions of functional macromolecular modules within migrating cells. This provides a means to coarse-grain the overwhelming molecular-scale complexity to a level that is tractable with imaging-based approaches, as recently demonstrated [16], [17] (Figure 1 A and B). However, even given such coarse-graining, it remains necessary to focus on a subset of selected modules that are central to the process of interest. In the case of (mesenchymal) cell migration, integrin-mediated cell-matrix adhesion complexes (CMACs – including focal adhesions, focal complexes and nascent adhesions) and the F-actin microfilament system are core macromolecular modules that directly mediate the migratory process (Figure 1 B) [8], [18], [19]. CMACs and F-actin serve as both mechanochemical-sensors and -transmitters, acting at the interface between cells and their environment to collect and functionalize information derived from extrinsic regulators in the distributed molecular network (Figure 1 A) [7], [20]. As such, their status is innately linked to, and therefore partially representative of, the aggregate informational state of the cell migration system as a whole [8].

Using live cell fluorescence imaging and quantitative image analyses, *organizational* features (morphological, positional, dynamical and compositional) of the cell migration system can be extracted to create a quantitative, multivariate characterization of CMAC and F-actin status on a per cell basis, as well as of cellular scale morphology (Figure 1 E). Simultaneously, *behavioral* features can be recorded describing the migration of the same individual cells (Figure 1 F). This facilitates two of the critical enabling capabilities of the research framework described herein, namely the: i) direct integration, and ii) temporal resolution of *organizational* and *behavioral* data on a per cell basis [21].

Firstly, direct data integration on a per cell basis allows the co-variance that arises between any two features as a result of natural cellular heterogeneity to define inter-feature correlations, making perturbations unnecessary to achieve this result. Crucially, this includes defining the relationships between features of cell *organization* and *behavior*. Secondly, the temporal resolution of variations in feature values allows us to move beyond correlative analyses to define the direction of information flow, in the form of causal influence, between pairs of system features using the Granger causality concept. Again, unlike many other systems-level causal analyses using, for example, network perturbation approaches [17], [22]–[25], directed causal influences can be defined using Granger causality without the need for perturbations.

The Granger causality concept originates from the field of econometrics [26], [27], wherein the study of causality cannot routinely be facilitated by perturbation-based analysis. Similarly, Granger causality has become a key tool in the perturbation-independent mapping of neural connectivity and information processing [28]. Herein we demonstrate one of the first implementations of this approach to the study of fundamental cell biology [15]. Essentially, the Granger conception of causality stipulates causation if the combination of past information from both a background variable, X, and a response variable, Y, improves the prediction of future values of Y, when compared to prediction of Y based on only its own past values. Such improved prediction implies the presence of unique, pre-emptive information in variable X and therefore its causal influence over variable Y.

We now apply a statistical implementation of the Granger causality concept to map directionally specific causal influences between pairs of recorded features (Figure 1 G). Thus we begin to define a wiring pattern for the unperturbed cell migration system. Subsequently, we employ well-characterized perturbations to reveal plasticity in this wiring pattern. This is a fundamentally important finding because it demonstrates that the perturbations frequently used to illuminate wiring patterns may, in some cases, partially distort our view of such information processing structures. We therefore propose that, for the purpose of mapping information flow in complex cellular systems, perturbation-independent strategies such as that described herein may provide a valuable complement to existing perturbation-dependent approaches.

## Results

### Imaging and Quantitative Analysis of Individual Migrating Cells

**Figure 2:**
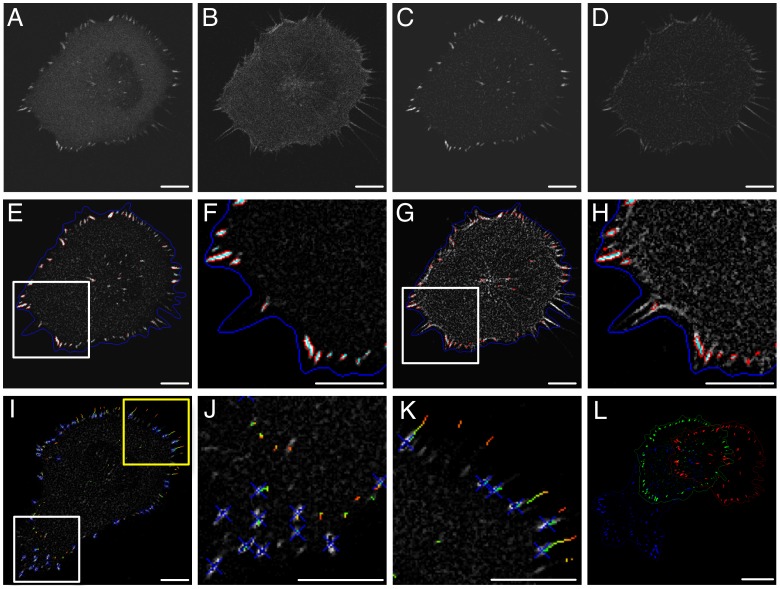
Imaging, segmentation and tracking of migrating cells and their CMACs. Confocal imaging of EGFP-Paxillin (A) and RubyRed-LifeAct (B) was performed simultaneously for 8 h at 5 min intervals (Supporting Movie S1 [left]). EGFP-Paxillin (C) and RubyRed-LifeAct (D) images were processed by median filtering and background correction. RubyRed-LifeAct signal was used for automated cell segmentation (dark blue outline in E–H). CMACs were segmented based on EGFP-Paxillin signals (red outlines in E, Supporting Movie S1 [center], and enlarged in F from white box in E). The RubyRed-LifeAct channel is shown overlayed by the EGFP-Paxillin-segmentation profile from E (G and enlarged in H from white box in G). Cells and CMACs were tracked via nearest neighbor analysis. CMAC tracking clearly differentiates stationary adhesions (I, enlarged in J from cell front (white box) in I) from sliding adhesions (I, enlarged in K from cell rear (yellow box) in I) (CMAC trajectories color coded for time, ≤10 time points shown, Supporting Movie S1 [right]). (L) Cell displacement over several hours (images from three time points, 0 h:20 min, 3 h:10 min, 5 h:50 min overlayed as red, green, blue, respectively). Quantitative variables describing cell and CMAC features/dynamics were automatically extracted (88 Single Cell Scale variables, Supporting Table S1, 29 variables defining individual CMACs, Supporting Table S2). Scale bars: A–K = 10 µm; L = 20 µm.

Live randomly migrating H1299 (human non-small cell lung carcinoma) cells stably expressing EGFP-Paxillin (CMAC marker) and RubyRed-LifeAct (F-actin marker) (H1299-P/L cells) were imaged by confocal microscopy. More than 4200 images were analyzed to segment and track individual cells, as well as their cohort of CMACs (totaling >70 000 CMACs) (Figure 2, Supporting Movie S1). We then extracted 88 quantitative features (Supporting Table S1) characterizing CMACs, their associated F-actin, cell morphology and cell migration on a per cell basis to produce a coarse-grained, synchronous characterization of cell migration system *organization* and *behavior* based on morphological, positional, dynamical and compositional features. Features encapsulate differing states of data aggregation in accordance with spatial and temporal data hierarchies (Supporting Figure S1). Experimental and analytic standardization ensured the consistency of quantitative data between, as well as within the time-course of, individual experimental repeats (Supporting Figure S2).

### Confirmation of a General Quantitative Link between Organizational and Behavioral Features

A critical premise of this study is that the quantitatively recorded CMAC, F-actin and cell morphological features should act as informative representatives of the *organizational* state of the broader cell migration system, and should therefore correlate on a per cell basis with the *behavioral* output of that system. We tested this premise to verify the basic relevance and informational value of our extracted *organizational* features by determining whether difference hierarchies, summarizing relative statistical distances between cell subpopulations, were equivalent for measures of *organization* and *behavior* (Supporting Figure S3). Importantly, we used two complementary methodologies, stratifying control cell subpopulations based on either their *organizational* (Supporting Figure S3 A–D) or *behavioral* (Supporting Figure S3 E–G) features. In both cases, we observed an ordinal equivalence between the difference hierarchies defined in *organizational* and *behavioral* data. These findings confirm the existence of a quantitative link between our *organizational* and *behavioral* measures, and further hint at the possibility of their functional correspondence.

### Selection of Organizational Features Associated with Cell Migration Speed

**Figure 3:**
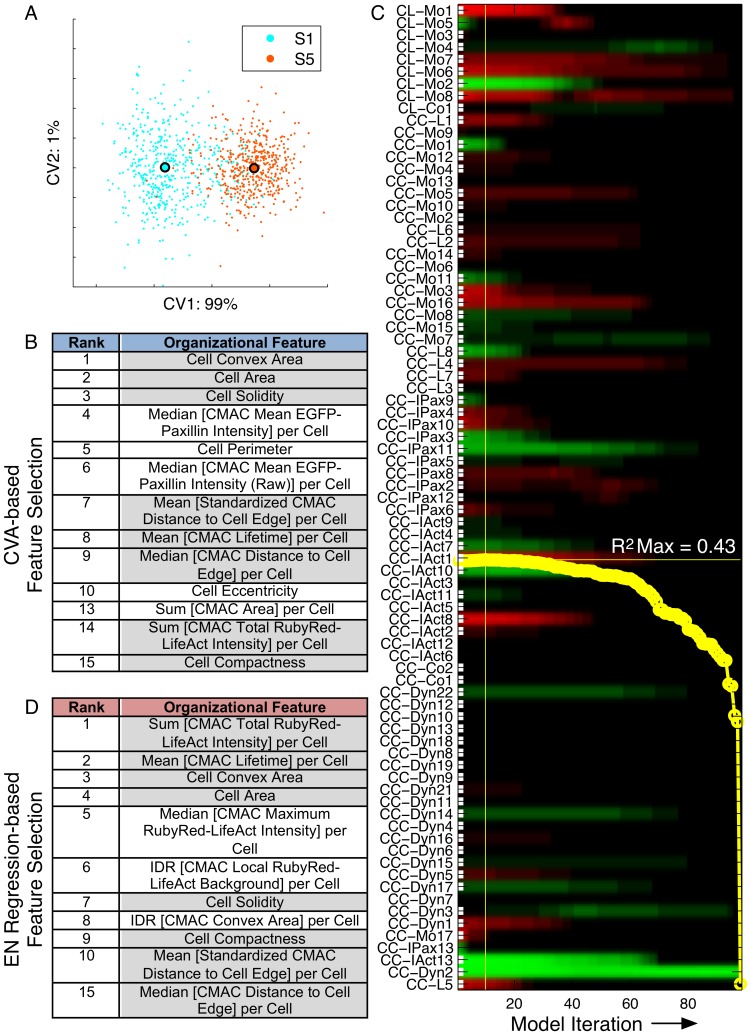
Selection of organizational features associated with Instantaneous Cell Speed (ICS). (A) Comparative CVA clustering of the slowest (S1, 1–20%, blue) and fastest (S5, 81–100%, red) moving *Instantaneous Cell Speed* (*ICS)*-defined cell subpopulations using all *organizational* variables achieves strong separation along the 1^st^ canonical vector (X-axis, captures 99% of total variation). (B) *Organizational* features driving separation of subpopulations selected by rank ordering according to absolute coefficient values associated with each feature in the 1^st^ canonical vector. (C) 81 normalized *organizational* features (background variables, Y-axis, compact feature names defined in Supporting Table S1) contribute to EN-regression modeling of *ICS* (*behavioral* feature, response variable) for the entire control cell population. Horizontal color bars indicate coefficient values (−1<x<1) (green = negative, red = positive, black = near zero) associated with each variable at any iteration (X-axis) of the modeling process. Red and green variables are positively or negatively correlated with *ICS*, respectively, while absolute coefficient values indicate the importance of each variable to the estimation of *ICS* per model iteration. With progressive model iterations, the sum of all coefficient values is forced non-uniformly towards zero, with coefficients redistributed to optimize the regression model according to adjusted R^2^. (D) Rank ordering of background variables based on their absolute coefficient values in the optimal regression model (adjusted R^2^ = 0.43, horizontal yellow line, iteration 10, vertical yellow line) provides a second list of *organizational* features associated with variations in *ICS*. Variables with grey backgrounds were implicated among the top 15 features by both feature selection methods (B and D).

We next used two independent feature selection methods to identify specific subsets of *organizational* features that were associated with variations in our main *behavioral* measure, *Instantaneous Cell Speed* (*ICS)*. Firstly, we used canonical vector analysis (CVA) to cluster the slowest (S1, 1–20%) and fastest (S5, 81–100%) moving quintiles of control cells using all *organizational* variables (Figure 3 A). CVA effectively separated these subpopulations along the 1^st^ canonical vector. Accordingly, ranking of variables based on their absolute loading coefficient in the 1^st^ canonical vector highlighted *organizational* features associated with the *behavioral* divergence between fast and slow migrating cells (Figure 3 B).

As an alternative feature selection method, we performed multiple regression via the elastic net (EN-regression) to estimate cell migration *behavior* (*ICS*, response variable) based on iteratively optimized combinations of 81 normalized *organizational* features (background variables, non-normalizable features were excluded to satisfy assumptions of ordinary linear regression). EN-regression was specifically selected for this task due to the enhanced stability of models produced in the presence of multicollinear background variables. The resulting EN-regression model achieved a maximal adjusted R^2^ value of 0.43, thus explaining 43% of the observed variability in *ICS* in a linear regression model (Figure 3 C). *Organizational* features included within the optimal regression model were ranked according to their absolute coefficient value, which indicates their individual contribution to the estimation of *ICS* (Figure 3 D).

Notably, comparison of the *organizational* features highlighted by both CVA- and EN-regression-based feature selection methods revealed more than 50% overlap among the high ranked predictions (Figures 3 B and D, respectively), leading to greater confidence in their potential importance. Conversely, disparities in selected features may be either methodologically driven or reflective of differences in input data, i.e. fast and slow control cell subpopulations (40% of cells, CVA method) versus all control cells (EN-regression method).

### Analysis of Correlations between Organizational Features and Cell Migration Speed

**Figure 4:**
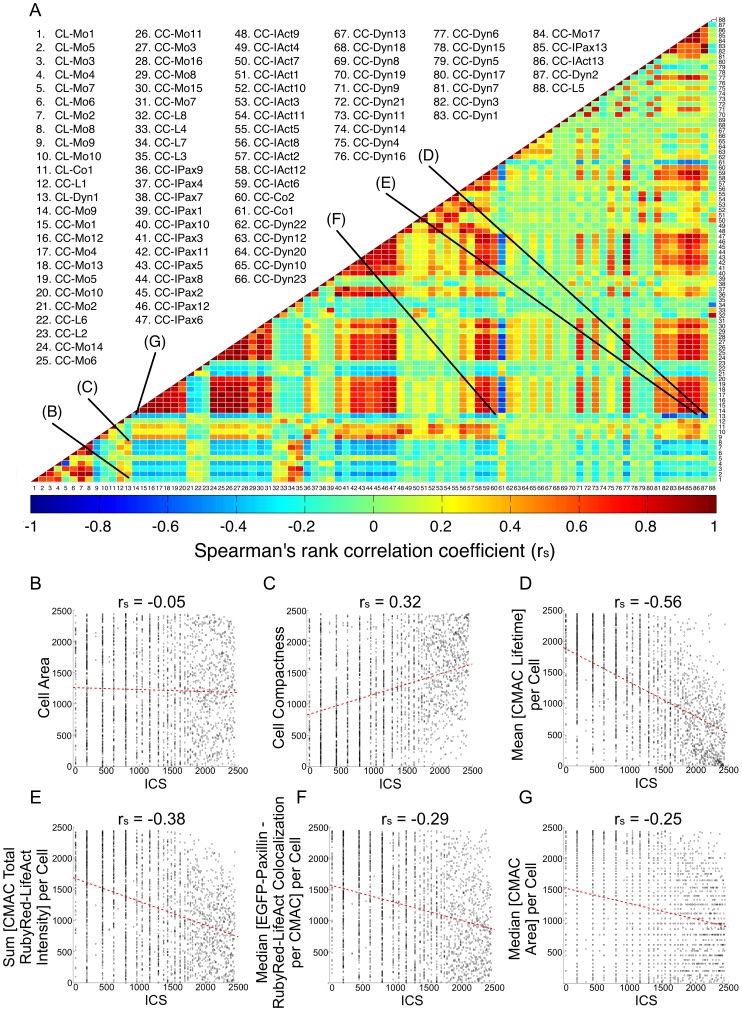
Exploration of individual feature correlations. (A) A heat map of Spearman’s rank correlation coefficients (rs) summarizes the pairwise correlative relationships between all 88 recorded variables (*organizational* and *behavioral,* compact feature names defined in Supporting Table S1) based on ranked observation values (blue = negative rs; red = positive rs; green = rs ∼ 0). (B–G) Selected correlations to ICS (indicated in heat map by lines (B)–(G), corresponding to panels B–G) plotted as ranked values of *ICS* (X-axes) vs ranked values of *organizational* features (Y-axes): *Cell Area* (B); *Cell Compactness* (C); *Mean [CMAC Lifetime] per Cell* (D); *Sum [CMAC Total RubyRed-LifeAct Intensity] per Cell* (E); *Median [EGFP-Paxillin – RubyRed-LifeAct Colocalization per CMAC] per Cell* (F); *Median [CMAC Area] per Cell* (G). Red dotted trend lines represent linear best fits.

We next analyzed Spearman’s correlation coefficients (r_s_) for pairwise combinations of all *organizational* and *behavioral* features (Spearman’s selected due to improved handling of non-linear correlations – similar values were obtained using Pearson’s correlation coefficient). Results are represented as a global inter-feature correlation heatmap (Figure 4 A). This summarizes the inter-feature relationships dictated by both mathematical and biological feature interdependencies. We then visualized the co-distribution of rank-ordered *ICS* values versus rank-ordered values of *organizational* features (Figure 4 B–G) chosen based on either the feature selection process described in Figure 3 or literature-based expectations. Ranked value visualization corresponds closely to Spearman’s correlation analysis. Interestingly, despite ranking highly according to both feature selection methods, *Cell Area* showed little or no correlation with ICS (r_s_ = −0.05, Figure 4 B). In contrast, *Cell Compactness* (r_s_ = 0.32, higher values indicate less round cells), *Mean [CMAC Lifetime] per Cell* (r_s_ = −0.56, values indicate the temporal stability of CMAC populations, per cell) and *Sum [CMAC Total RubyRed-LifeAct Intensity] per Cell* (r_s_ = −0.38, values indicate the total enrichment (after local background subtraction) of RubyRed-LifeAct signal within CMACs, per cell) all showed strong correlations to *ICS* (Figure 4 C–E). *Median [EGFP-Paxillin – RubyRed-LifeAct Colocalization per CMAC] per Cell* (r_s_ = −0.29, values indicate the median Pearson’s correlation between F-actin and CMAC marker signals within segmented CMACs, per cell) also showed a negative correlation to *ICS*, as did *Median [CMAC Area] per Cell* (r_s_ = −0.25, values indicate the median area of individual CMACs, per cell). This final correlation between adhesion size and cell speed is in agreement with recent findings [16].

### Identification of Directed Causal Interactions in Cell Migration

To extend upon the directionally unspecific correlative indications derived via CVA, EN-regression and Spearman correlation analyses, we next engaged the Granger causality concept [26], [27]. This permitted detection of the directional flow of information, as revealed by causal influence, between individual features defining cell migration system *organization* and *behavior*. Specifically, we performed Granger causality modeling via auto-regression to determine how the values of background (*organizational*) variables at preceding time-points (temporal lags) contributed to the prediction of future values of the *behavioral* response variable, *ICS*. These analyses focused initially on those variables previously highlighted via correlative analyses (Figures 3 and 4).

**Figure 5:**
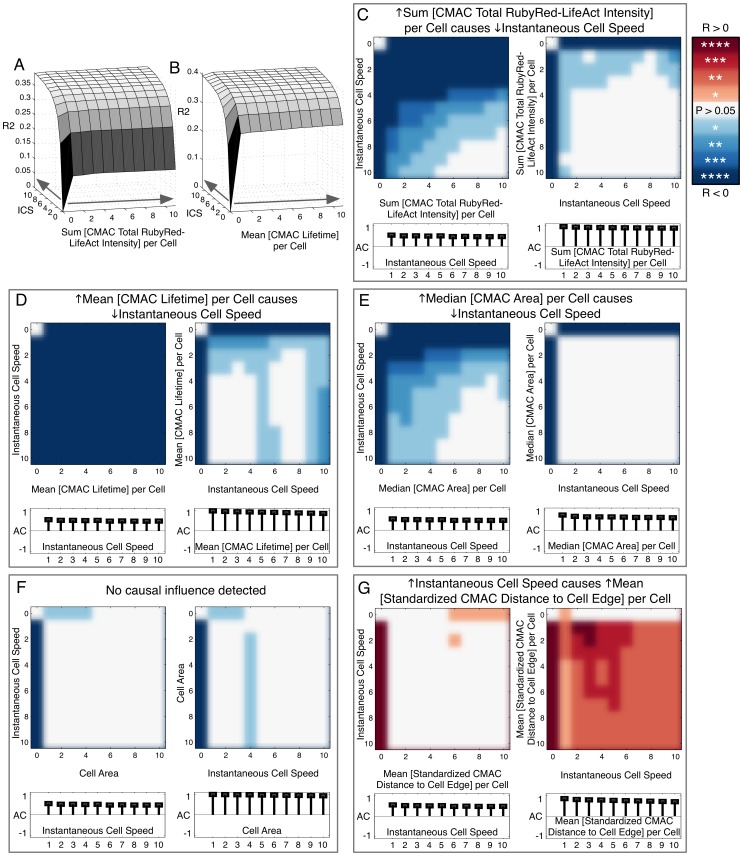
Mapping of directed causal influence based on Granger causality. (A and B) 3D surface plots of adjusted R^2^ values (Y-axes) based on auto-regression (AR) modeling of the response variable, *Instantaneous Cell Speed (ICS)*, using combinations of one to ten temporal lags (5 min interval) of the response variable (*ICS*, Z-axes) and one to ten temporal lags of a background variable, either *Sum [CMAC Total RubyRed-LifeAct Intensity] per Cell* (X-axis, A) or *Mean [CMAC Lifetime] per Cell* (X-axis, B). Grey arrows along X and Z axes indicate the inclusion of additional temporal lags of the indicated variable (A and B). (C–G) Significance testing of improvements in adjusted R^2^ values caused by the addition of temporal lags of background (X-axes) and response variables (Y-axes) to an AR model based on >2200 cell observations. White indicates no statistically significant improvement in prediction. Blue and red color schemes (indicating negative and positive correlations between background and response variables, respectively) are each divided into 4 levels of significance, with P values <0.05 (*), <0.01 (**), <0.001 (***) or <0.0001 (****), as indicated (color scale-bar, upper right). Causation is tested reciprocally within variable pairs to discern evidence for the causal influence of *organizational* variables over *ICS* (left panels, C–G) and *vice versa* (right panels, C–G). We infer causal influence only where significance-testing patterns are robust and ordered (as in C [left panel], D [left panel], E [left panel], G [right panel]. Stem plots (below X-axes, C–G, left and right panels) indicate the degree of autocorrelation in respective response variables.

As shown in Figure 5 A, auto-regression modeling indicated that progressively adding temporal lags of *ICS* alone increased *ICS* prediction accuracy to a maximal adjusted R^2^ of ∼0.4. Thus, current *ICS* is dependent on past *ICS*. Notably, as implied by the preceding correlative analyses, temporal lags of the *organizational* features *Sum [CMAC Total RubyRed-LifeAct Intensity] per Cell* and *Mean [CMAC Lifetime] per Cell* each alone enabled (partial) estimation of future *ICS* (adjusted R^2^ max 0.12 and 0.28, respectively, Figure 5 A and B). Such results indicate either *correlation* and/or *causation* between these *organizational* features and *ICS*. Crucially, explicit evidence of Granger *causation* was achieved by combining lags of *ICS* with lags of these background variables, resulting in clear improvements in adjusted R^2^. Thus, both *Sum [CMAC Total RubyRed-LifeAct Intensity] per Cell* and *Mean [CMAC Lifetime] per Cell* contributed additional information to the prediction of *ICS*, implying association with up-stream Granger causal mechanisms.

We next assessed whether the improvements in prediction provided by background variables were statistically significant over a population of >2200 control cell observations. Impressively, the addition of information from either *Sum [CMAC Total RubyRed-LifeAct Intensity] per Cell* (Figure 5 C, left panel) or *Mean [CMAC Lifetime] per Cell* (Figure 5 D, left panel) conferred statistically significant improvements in adjusted R^2^ values for the majority of background and response variable lag combinations. Thus, we conclude that these variables Granger cause *ICS*. Specifically, given the negative correlations observed, increases in CMAC lifetime and total RubyRed-LifeAct associated with CMACs both inhibit *ICS*. Note that the degree of autocorrelation in response variables does not correlate with the ability to detect causal interactions.

Because causal interactions may be bi-directional, we also tested for Granger causation in the reverse direction. As shown in Figures 5 C (right panel) and D (right panel), lags of *ICS* could not consistently improve the estimation of future values for the two selected *organizational* features. Combined, we interpret these results as indicating predominantly uni-directional Granger causation *of Sum [CMAC Total RubyRed-LifeAct Intensity] per Cell* and *Mean [CMAC Lifetime] per Cell* on *ICS* and not *vice versa*. Similarly, causality analysis of *Median [CMAC Area] per Cell* also indicated that increasing values of this variable Granger caused the inhibition of *ICS* (Figure 5 E, left panel), and again, no Granger causation could be detected from *ICS* towards *Median [CMAC Area] per Cell* (Figure 5 E, right panel).

Significantly, consistent with our correlative analyses, *Cell Area* data did not improve the estimation of future *ICS*, and nor did past *ICS* improve estimation of future *Cell Area* (Figure 5 F, left and right panels, respectively). Thus, no evidence for Granger causation could be discerned between these cellular features. In a surprising contrast, analysis of *Mean [Standardized CMAC Distance to Cell Edge] per Cell* (values indicate average CMAC proximity to the nearest cell edge as a proportion of the distance from the cell edge to cell center of area, per cell) provided no evidence for Granger causation on *ICS* (Figure 5 G, left panel), but instead was Granger caused by *ICS* (Figure 5 G, right panel), such that faster cell migration drives a more centralized distribution of CMACs within the cell.

### Identification of a Chain of Causation in Cell Migration

**Figure 6:**
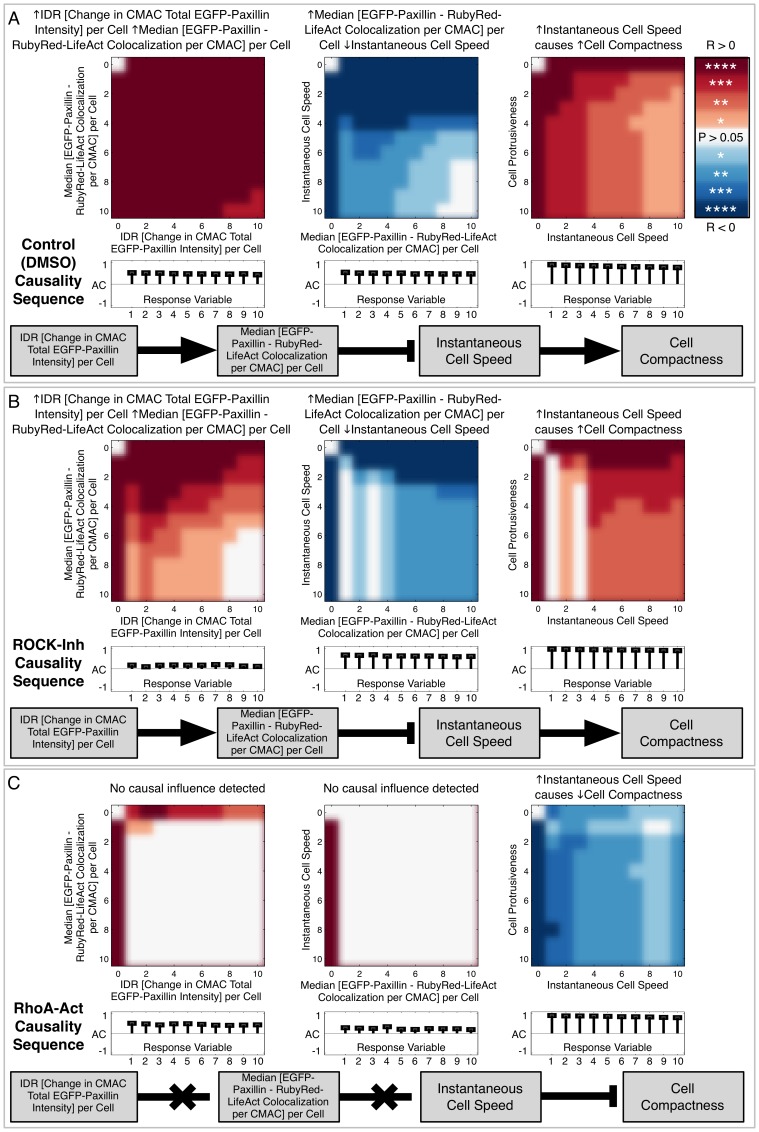
Causal influence patterns are plastic and contextually dependent. (A) Granger causality analysis revealed a sequence of causal interactions extending both up-stream and down-stream of *Instantaneous Cell Speed (ICS)*: Increasing *IDR [Change in CMAC Total EGFP-Paxillin intensity] per Cell* (indicates per cell variation in the net rate of EGFP-Paxillin recruitment/release per CMAC) caused increased *Median [EGFP-Paxillin – RubyRed-LifeAct Colocalization per CMAC] per Cell* (A, left panel). Increasing *Median [EGFP-Paxillin – RubyRed-LifeAct Colocalization per CMAC] per Cell* caused reduced *ICS* (A, center panel). Increasing *ICS* caused increased *Cell Compactness* (A, right panel), indicating that fast moving cells become less round. The causal links between these four variables are summarized schematically (grey boxes), with positive and negative relationships indicated by arrows and capped lines, respectively. Analyses of Granger causality predictions for equivalent inter-feature relationships in ROCK-inhibited cells (B) reveal comparable causal relationships while Rho-activated cells did not (C). Notably, although the final causal relationship (C, right panel) was still detected, the causal effect was reversed such that increasing *ICS* caused decreasing *Cell Compactness,* i.e. increased cell speed caused cells to become more round. All variables are defined in Supporting Table S1.

We next explored the causal wiring pattern of cell migration more comprehensively by examining an extended set of inter-feature relationships. This revealed interconnected patterns of Granger causation extending beyond single pairs of variables. As an example, we present a short chain of Granger causal interactions extending both up- and down-stream of *ICS* (Figure 6 A). This sequence begins with the variable *IDR [Change in CMAC Total EGFP-Paxillin intensity] per Cell* (values indicate per cell variance in the net rate of change in EGFP-Paxillin content per CMAC). Increases in this variable enhanced *Median [EGFP-Paxillin – RubyRed-LifeAct Colocalization per CMAC] per Cell*, which, as noted previously, signifies the Pearson’s correlation between paxillin and F-actin protein concentrations within CMACs. Such enhancement Granger caused reduced *ICS* (Figure 6 A, center panel). Finally, *ICS* Granger caused changes in cell shape, as represented by *Cell Compactness* (a size-independent measure of cell roundness), such that higher *ICS* promoted less rounded cells with higher *Cell Compactness* values.

### Differential Perturbation Reveals the Plasticity of the Defined Causal Wiring Pattern

To assess the question of whether causal wiring patterns are stable and generalizable, or plastic and contextually dependent, we assessed the robustness of the Granger causality chain described in Figure 6 A using data derived from cells perturbed via either the inhibition of ROCK or the activation of Rho (Supporting Movies S2 and S3). These represent treatments with strong, well-characterized impacts on cell migration. The effects of these perturbations were extensively assessed (Supporting Figure S4), revealing excellent coincidence with previous observations. Remarkably, despite dramatically altering both cell migration system *organization* and *behavior*, inhibition of ROCK did not significantly disrupt the chain of Granger causality first detected under control conditions (Figure 6 B), thereby providing additional support for the significance of these findings. In contrast, Rho activation disrupted the first two causal relationships such that no evidence of Granger causation could be detected (Figure 6 C). Strikingly, the presence of the third causal relationship was conserved following Rho-activation, but the impact of the relationship was reversed such that increasing *ICS* reduced, rather than increased, *Cell Compactness*. These results, particularly the positive evidence indicating inversion of effects in the third interaction, reveal the differential sensitivity of causal interactions to perturbation and, more generally, their contextual dependence. Fundamentally, this exposes plasticity in the causal wiring pattern underlying the cell migration process.

## Discussion

A variety of studies have previously described correlations between *organizational* and *behavioral* features of cell migration, yet the orientation of cause and effect in these relationships has remained undefined [16], [29]–[33]. We now extend on such correlative analyses by describing a novel strategy for directionally specific mapping of information flow in the form of causal influence. A key differentiating aspect of our approach is the focus on charting causal influences based on sensitivity to the natural heterogeneity (specifically, time-resolved inter-feature co-variance) that arises *within individual* experimental conditions, as opposed to relying on perturbation-induced differences between conditions. This permits: a) the delineation of causal influence patterns on a per condition basis, which in turn facilitates; b) the comparison of these wiring patterns between alternatively perturbed conditions. Through these capabilities we now provide proof-of-principle for plasticity and contextual-dependence in the regulatory wiring of the cell migration system, while also delineating specific, functionally significant relationships between key features within this system.

The plasticity of causal wiring in the cell migration system is illuminated by differences observed in pair-wise causal influences under control (DMSO-treated) and Rho-activated conditions. Specifically, this plasticity is evidenced by the disruption (or weakening to undetectable levels) of two causal interactions within the causality chain defined in control cells, and is positively exemplified by alterations to the causal interaction detected between cell speed and cell compactness. The nature and implications of these specific alterations are discussed further below. In general, however, these perturbation-induced differences in inter-feature connectivity provide proof-of-principle as to the flexibility and adaptability of the causal wiring pattern governing cell migration. Concomitantly, the equivalence between the causality chains revealed in control and ROCK-inhibited cells strongly support the validity and relevance of the defined causal interactions. Thus, in combination, these unique findings demonstrate both robustness and contextually dependent plasticity in the functional relationships between coarse-grained features at the core of the cell migration system. These findings hold the important implication that although some causal regulatory influences may be conserved even under strongly altered conditions, a singular characterization of inter-feature relations is unlikely to be generalizable. This notion seems likely to also be important for the molecular-scale networks and components that underpin the coarse-grained macromolecular features assessed herein.

It should be noted that the concept of plasticity is already well established in the context of cell migration behavior [2]. However, such behavioral plasticity may arise mechanistically from two distinct types of variation within the underlying network: 1) variation in the properties of features (nodes) within the network, and/or; 2) variations in the connectivity (edges) between those features. To date, numerous studies have described how specific perturbations induce changes in the values of features-of-interest within the cell migration system. In notable examples, extensive arrays of F-actin, CMAC and cell behavioral properties were monitored and compared across a spectrum of conditions wherein ECM-density, intracellular contractility and/or growth factor stimulus were modulated [29], [30]. Although important correlative links were implied by these studies, direct quantitative correlations between many of the recorded features were inaccessible because data were derived from distinct experimental sources. This highlights the advantages of defining inter-feature connectivity based on multivariate data derived from within individual experimental sources and, indeed, from within individual conditions (rather than by comparison between conditions). Such strategies rely on sensitivity to natural heterogeneity *within* conditions, thereby allowing the detection of variations in the inter-feature connectivity *between* conditions. A highly relevant study where this was achieved includes an analysis of how correlative connections between CMAC morphology-features were altered by individual siRNAs within an RNAi screen [32]. Indeed, this study is particularly noteworthy because it documents plasticity in the *correlative* relationships between morphological CMAC features. Our work now builds on elements of this important foundation in two ways: 1) assessment of the relationships between features of cell migration system *organization* and *behavior*, as facilitated by synchronous acquisition of both data types on a per cell basis, and; 2) definition of the direction of causal influences between features enabled by time-resolved data and Granger causality analysis.

The extension of correlative analyses to delineate causal influence marks a significant advance. As noted in a recent commentary, the majority of analyses in cell biology are correlative, with the analysis of causation remaining infrequent [15]. This is particularly true at a systemic level where complex patterns of inter-feature causation are considered. However, one prominent strategy for empirically defining causal wiring patterns is network perturbation [17], [22]–[25]. Generally this approach involves the characterization of an array of features in both the presence and absence of perturbation(s). The variation in feature values enforced by perturbations is then used to detect connectivity between feature-pairs and, specifically, inter-feature causation. Although extremely powerful, some limitations to the network perturbation strategy may bear further consideration here. Firstly, as with more locally focused epistasis strategies, inter-feature connectivity is defined by comparison of feature values between control and perturbed conditions. Because the *combination* of conditions is used to define a *single* causal wiring pattern, network plasticity cannot easily be addressed through the comparison of wiring patterns. A noteworthy exception to this is the time-resolved network perturbation analysis performed by Ku *et al*, which demonstrated the temporal evolution of causal influence patterns between coarse-grained features of microtubule, F-actin and myosin machineries during neutrophil polarization [17]. A second limitation associated with the network perturbation strategy is that the required perturbations are assumed to alter feature values (to illuminate inter-feature connectivity) *without* distorting the “native” network architecture, or at least to do so only locally at the expected point of perturbation [34]. Crucially, our detection of plasticity in causal wiring following Rho activation provides proof-of-principle that this assumption may not always hold. Thirdly, in a critique common to many perturbation-dependent strategies, the specificity of targeted perturbations is often fundamental to the interpretation of causal directionality within inferred wiring patterns. Of similar importance is the relative timing of direct and indirect effects that propagate downstream of the initial perturbation, including possible feedback responses. Where these are not clearly characterized, appropriate inference of causal directionality involves assumptions that, although reasonable, may not always be satisfied. Thus, overall, it seems important to recognize that limitations exist in relation to the use of perturbations for the definition of causality, either locally (as in epistasis) or at a network level. Though these perturbation-based approaches are clearly valuable and indispensable, their limitations in some cases coincide with relative strengths of the novel strategy described here, wherein perturbations are not required for the coarse-grained definition of “native” causal influences. Thus, perturbation-independent strategies such as this one may provide a useful complement to existing approaches.

The detection of causal wiring pattern-plasticity is a key finding, but the individual inter-feature relationships defined herein each also hold their own specific relevance. For example, the association between F-actin and CMACs is thought to be a critical determinant of migratory behavior [20]. Our finding that increasing RubyRed-LifeAct signal (representing F-actin) associated with CMACs causes decreased cell speed resolves the direction and effect of this previously correlative indication. Similarly, correlations have also been shown between CMAC stability (represented by lifetime) and cell speed [29]–[31]. We now confirm existing assumptions regarding the inhibitory causal influence of high CMAC stability over cell speed. Likewise, our finding that increasing CMAC size inhibits cell speed is consistent with the correlative indications of a recent study that used aggregate inter-feature co-variance enforced by parallel perturbations to define correlations between CMAC features and cell migration [16]. Interestingly, we reveal that a variety of CMAC features have causal influence over cell speed, while Kim *et al* found that only CMAC area (of several assessed CMAC features) robustly correlated to cell speed. One explanation for this difference may be that causal wiring plasticity induced by each perturbation (as demonstrated herein) may result in an unintentional stringency within the Kim *et al* study, such that only those correlations conserved under all conditions would be detected in the final analysis. This emphasizes the value of a perturbation-independent strategy for wiring pattern-definition.

While the three causal relationships noted above corresponded to existing expectations based on previous correlative analyses, the findings for cell area, CMAC localization and cell shape were less expected. Firstly, no evidence was detected that cell area has any causal relationship with cell speed. This was unexpected both because cell area globally modulates intracellular signaling networks [35], [36] and because numerous perturbations impact coincidently on cell migration and cell area, leading intuitively to inference of a causal dependency. While the absence of evidence for a causal interaction is not proof that no such relationship exists, it might reasonably be interpreted as indicating either the absence or relative weakness of an interaction. This is interesting in the light of our feature selection results wherein cell area was consistently indicated to be an important parameter. This dichotomy may be explained by recognition of the fact that our correlative and Granger analyses were pairwise, while feature selection methods were multivariate. Whereas such pairwise analyses have sensitivity for strong biological relationships that are relatively direct and linear, multivariate analyses may provide sensitivity towards complex and perhaps contingent relationships between regulatory features. Thus, it is conceivable that cell area may modulate the causal impacts of several other features upstream of cell speed, in such a way that its own causal influence is distributed and not directly discernable. It will therefore be crucial in the future to develop multivariate statistical implementations of Granger causality analysis for this setting, including improving sensitivity to non-linear causal interactions [37].

Even more surprising was that both CMAC localization and cell shape features were regulated by cell speed. In general, these findings highlight ongoing questions regarding the possible orientations of causation relative to complexity in such complex biological systems [38], and indeed about the relative positions of such features in a hierarchy of complexity [9], [10]. Specifically, we were surprised in the first instance to find that faster cell migration caused increased distances between CMACs and the cell edge, reducing the peripheral skew in CMAC localization [39]. This was unexpected because our empirical findings refute previous perturbation-derived inferences that CMAC centralization may cause the inhibition of cell migration [40], [41]. Indeed, when combined with evidence that CMAC lifetime causally regulates cell speed, this finding allows the disentanglement of previously convoluted correlations between CMAC centralization, CMAC stabilization and cell migration. Thus, we find that CMAC stability strongly influences cell speed, which in turn modulates CMAC localization.

Equally unexpected was the finding that cell speed lies upstream of cell shape determination, with faster migration in control cells promoting a less round cell shape (with higher compactness values). While the existence of a correlative relationship between cell shape and cell speed has been abundantly clear [42], [43], the direction of causation has not. However, evidences that symmetry breaking and associated cell deformations necessarily precede cell migration [44] promote the assumption that cell shape would act upstream of cell speed, rather than downstream as found here under all three experimental conditions. A further investigation of the relative magnitude and frequency of shape changes during symmetry breaking and at different migration speeds may more fully explain this surprising finding. In general, this observation reveals a possible limitation in the sensitivity of our causal analysis strategy, since each experimental conditions is described by a single causal wiring pattern. This means that potential variations in causal wiring arising within conditions may be lost. It is conceivable, for example, that wiring patterns may differ during alternate phases of migration (symmetry breaking, protrusion, retraction) and/or between different migratory modes (mesenchymal vs amoeboid). Indeed, the latter is hinted at by wiring changes induced following Rho-activation, as discussed further below. Methodologically, the ability to disaggregate data from within experimental conditions to identify recurring migratory phases/modes may help to contextualize our understanding of causal network structures during evolving migratory behaviors, as previously exemplified during cell polarization [17].

As noted above, the causal influence of cell speed over cell shape revealed in control cells was recapitulated in both ROCK-inhibited and Rho-activated cells. This is remarkable given the dramatic alterations to *organizational* and *behavioral* features induced by each of these perturbations. However, the inversion of the causal effect of this interaction in Rho-activated cells is particularly noteworthy, and not only as proof-of-principle for causal wiring pattern-plasticity. Indeed, the finding in Rho-activated cells that higher cell speed causes cells to become rounder, rather than more protrusive, is reminiscent of a switch in the organizational state of migratory machinery from a mesenchymal phenotype towards an amoeboid phenotype. This is a known effect of Rho activation [2]. Such a switch in migratory mode is further supported by a comparison of the quantitative changes recorded in our Rho-activated cells and those observed following Rho-activation in MDA-MB231 cells in 2D culture by means of Smurf1-depletion [45] – a perturbation which drove a pronounced mesenchymal-amoeboid transition in 3D culture. Thus, our analysis of causality following Rho-activation reveals not only plasticity in causal wiring, but that this plasticity may correspond to a recognized switch between mesenchymal and amoeboid modes of migratory behavior.

The observation that higher variation in the rate of net EGFP-Paxillin incorporation/release from CMACs is predictive of increased intra-CMAC colocalization between EGFP-Paxillin and RubyRed-LifeAct is also noteworthy. It is consistent with demonstrations that accelerated paxillin dynamics correlate with high force application to CMACs [46], [47], which in turn correspond to CMAC maturation and reinforced associations with F-actin [48], [49], an effect observable via colocalization analysis [33]. Remarkably, it is specifically the variance in EGFP-Paxillin dynamics that is causal, since the median rate of EGFP-Paxillin incorporation/release at CMACs shows no evidence of causation. This highlights the potential for population variance to have causal influence independent of mean/median values – an information processing feature that may be associated with relative rather than absolute signal processing. Finally, the finding that increasing colocalization between EGFP-Paxillin and RubyRed-LifeAct within CMACs causes inhibition of cell migration is in correspondence with previous correlative findings showing that increased CMAC-component-to-F-actin colocalization coincides with force-driven CMAC maturation and reduced cell migration [29], [30].

In summary, we have presented a new imaging-based strategy designed to detect causal influence transmission between coarse-grained features synchronously characterizing the core machinery and behavioral outputs of the cell migration system. This approach employs the Granger causality concept to leverage natural heterogeneity within experimental conditions, facilitating perturbation-independent causal analyses. This is, to the best of our knowledge, the first time the Granger causality concept has been employed within such a cell biology setting. Using this approach, we have mapped several pair-wise causal interactions between features of cell *organization* and *behavior,* thereby alternately confirming or refuting existing assumptions of causation derived from previous correlative analyses. Most importantly, we have provided empirical evidence of plasticity in the causal wiring pattern underlying cell migration. This finding indicates that causal analyses should be performed and interpreted in a contextually dependent manner, since global causal wiring patterns are unlikely to be entirely generalizable.

Ultimately, this work provides new insights into the architecture of causal interactions that underlie cell migration, but more generally also presents a highly extensible framework for further exploration of this process. Future developments in experimental design, data acquisition (including an emphasis on molecular-scale information), and Granger causality analysis methods are likely to deliver important insights into cell migration, as well as the potential to explore a variety of complex, dynamic and heterogeneous cellular processes.

## Materials and Methods

### Cell Culture and Experimental Conditions

H1299 (human non-small cell lung carcinoma, ATCC) cells were transfected with both EGFP-Paxillin and RubyRed-LifeAct constructs using Lipofectamine 2000 (Gibco) according to the manufacturers instructions. Double-expressing stable clones (H1299-P/L) were selected and maintained in RPMI 1640 medium (Gibco) containing 1 mg/ml Geneticin (G-418 sulfate, Gibco), supplemented with 1 mM Glutamine and 10% fetal bovine serum (Gibco) at 37°C, 5% CO_2_. Fibronectin was purified from human plasma as described previously [50]. Imaging substrates were coated with purified Fibronectin (10 µg/ml) at 37°C for 2 h followed by blocking with 1% heat denatured bovine serum albumin (Sigma-Aldrich) at 37°C for 1 h. H1299-P/L cells were serum starved for 24 h prior to imaging. 4000 cells were plated per well in fibronectin-coated 96-well glass-bottomed plates (0.17 mm optical glass, Matrical Bioscience). Cells were treated immediately with DMSO (Di-methyl sulfoxide, Sigma-Aldrich) as control, or alternatively with either a Rho-associated protein kinase (ROCK)-inhibitor (Y-27632; 25 µM, Sigma-Aldrich), or a Rho activator (Calpeptin, 25 µg/ml, Cytoskeleton). In each experiment, control (DMSO-treated, 9 experimental repeats) cells were paired with one perturbed condition (ROCK-inhibition, 5 experimental repeats or Rho-activation, 4 experimental repeats) to allow subsequent comparison of results between these three conditions. Live cell imaging commenced after 2 h with treatments ongoing.

### Confocal Live Cell Fluorescence Imaging

Live cell imaging was performed on a Nikon A1R confocal microscope with a PlanApo VC 60X/1.4 NA oil-immersion objective. Images were acquired at 5 min intervals for 8 h with a pixel resolution of 0.21 µm. Cells were maintained during imaging in normal culture medium absent fetal bovine serum, *at 37°C and 5% CO_2_.*

### Image Processing and Image Analysis

Acquired images were processed in ImageJ (W.S. Rasband, NIH) using the Hybrid 3D median filter followed by local background subtraction. Processed images were then analyzed using PAD software (v6.3) (Digital Cell Imaging Laboratories, Keerbergen, Belgium). Cell boundaries were detected based on Ruby-LifeAct fluorescence, with cells excluded when contacting image borders. CMACs with area >0.05 µm^2^ were then segmented based on EGFP-Paxillin signal. Segmented Cells and CMACs were tracked over time based on nearest neighbor analysis. Cell and CMAC segmentation and object tracking were verified by direct visual inspection of all images and image sequences. Quantitative features defining cells, per Cell CMAC populations (Supporting Table S1) and individual CMACs (Supporting Table S2) were then automatically extracted. Intensity values within CMACs were corrected in each channel for local background within a 1 µm radius around the boundary of each CMAC, excluding signal from any other segmented CMAC.

### Data Parsing

Automated data parsing was performed prior to extensive statistical analyses. This included the removal of CMACs observed in only a single time point, thus dramatically limiting misclassification of noise as CMACs, especially given imposed limits on the maximal inter-time point displacement of CMACs (2 µm). Similarly, CMACs with minor axis values of less than a single pixel (210 nm) were removed to exclude aberrant form-factor calculations. CMACs present in the first and final images of an image sequence were excluded from aggregate calculations of CMAC Lifetime due to the biasing effect associated with the inclusion of incomplete CMAC lifetimes.

### Data Standardization

CMAC fluorescence intensity data was standardized by calculating the median of all individual CMAC mean intensities, per recorded channel, per experimental repeat in the control (DMSO-treated) condition, of CMACs between 0.15 and 0.2 µm^2^. This size range incorporates numerous CMACs and is above both the optical and digital resolution of the acquisition system, limiting ambiguity about actual CMAC area. All CMAC fluorescence intensity values per experimental repeat were standardized to this value.

### Data Transformations

Cell trajectories were smoothed through the application of smoothing splines. The parametrization of the smoothing was based on a trade off between maintaining the properties of the original trajectories (essentially no difference can be observed between original and smoothed trajectories), and enhancing the continuity of the instantaneous cell speed distribution. This diversification of ICS values enhanced the efficacy of subsequent statistical analyses (e.g. regression techniques).

Given our usage of ordinary least squares estimations in auto-regression and elastic net-regression, and the associated assumptions of residual normality, Box-Cox transformations [51] with individualized tunings were applied to normalize data distributions for each cell migration system feature, so as to fulfill this necessary assumption.

### Subpopulation Discovery via the EM-algorithm

Principal component analysis (PCA) using singular value decomposition of the normalized data matrix was first applied to control cell observations based on all 87 *organizational* variables. Subsequently, the first 25 principal components (containing 87.5% of total variation) were used in an expectation maximization (EM)-algorithm [52] for assignment of control cell observations to quantitatively defined subpopulations. EM algorithm-based assignments were repeated 10 times each for designation of between 2 and 8 subpopulations. The Akaike information criterion (based on the log-likelihood of the mixture model penalized by the number of existing groups) [53] was used to identify the optimal number of subpopulations. This number was found to be 4 (Figure 2). The final assignment of control cell observations between four subpopulations was then determined according to the lowest Akaike information criterion achieved after 100 iterations of the EM-algorithm (using the same data as above). Importantly, the EM-algorithm was initiated using random seeding so as to relieve the locking effect of initiating the algorithm with clustering techniques such as *k*-means clustering.

### Comparison of Defined Cell Subpopulations

Canonical vectors analysis (CVA) [54] was performed using decompositions of the between-group and within-group covariance matrices based on eigenvalue decomposition. Mahalanobis distance measurements were applied to determine the pairwise between-group distances using their structures of covariance.

Agglomerative hierarchical clustering of the 87 dimensional *organizational* data was performed on the mean vectors of subpopulations, subject to the shortest distance using the Euclidean distance as a metric. Notably, mean vectors were based on principal components of the multivariate data matrix. Outputs were displayed as standard dendograms.

Multivariate analysis of variance (MANOVA) was applied to the 87 dimensional data matrix to assess whether the between-group and within-group covariance matrices differ significantly given specific subpopulation assignments. Significance was determined via Wilk’s test.

Pairwise univariate distribution tests were performed using the two-sample Kolmogorov-Smirnov (KS)-test to determine whether two empirical probability density functions were likely to have arisen from the same underlying probability function. Significance was determined based on comparison of between-distribution KS-scores to the KS-test’s critical values at the 5% significance level. Given KS-scores beyond the significance level, individual variable distributions were visualized as probability distribution functions. These were estimated using kernel density estimation with Gaussian kernels and fixed bandwidths.

### Feature Selection

CVA was applied to find the *organizational* features that contribute to the separation of the fastest (5^th^ quintile of ICS) and slowest (1^st^ quintile of ICS) cells (DMSO-treated). Features were selected based on their absolute contribution to the first canonical vector which contained >90% of the between-group variation needed to maximally separate these subpopulations.

Regression via the elastic net (EN-regression) [55] was employed using all control cell observations, with ICS as the response variable (representing *behavior*) and *organizational* features as background variables. As noted previously, in order to meet the assumptions of the regression model, all variables were normalized via individually tuned Box-Cox transformations. After supervision, 6 variables were discarded (as non-normalizable) and the regression model was built using the remaining 81 *organizational* features as background variables. For feature selection, features were ranked based on their absolute contribution (coefficient) at the model iteration yielding the optimal goodness-of-fit along the shrinkage path of the EN-regression (as adjudged by the adjusted coefficient of determination (adjusted R^2^)). The decision to use EN-regression (as opposed to related techniques such as Ridge regression or the LASSO) was based on its grouping effect wherein the coefficients assigned to multicollinear (related) variables are not shrunk independently. The use of the adjusted coefficient of determination was motivated by our interest in the descriptive and not the predictive accuracy of the model.

### Analyses of Granger Causality

The concept of Granger causality [26] can be expressed in terms of auto-regression where a variable X is stated to Granger-cause another variable Y if lags of X, in the presence of lags of Y, improve the auto-regression model’s explanatory power of ‘present’ values of Y. More specifically, let us have two models A and B, where A has Y as the response variable and lags of Y as the background variables, and B has Y as the response variable and lags of *both* Y and X as the background variables. Then, given a significant reduction in the residual sum of squares of model B compared to model A, X is said to Granger-cause Y. Our analyses of Granger causality were performed on this basis using linear auto-regression. It is important to observe that as “older” lags are included into each regression model, “younger” lags are also retained, such that if a lag from 15 minutes prior to the “present” is included, the 5 and 10 minute lags are also included in the model. As noted above, all variables used in these analyses were normalized via individually tuned Box-Cox power-transformations. Furthermore, to confirm the stationarity of our data, we applied the augmented Dickey-Fuller test. This indicated that our time-series data did not contain significant trends such as fluctuating means or variances. Notably, data stationarity is necessary to satisfy the assumptions of ordinary least squares-based estimation and therefore the auto-regressive modeling applied in our assessment of Granger-causality. Finally, to test for significant improvements in model fitting (corresponding to the addition of the candidate causal variable) we utilized the Granger-Sargent test.

## Supporting Information

Figure S1 **Spatial and Temporal Data Hierarchies.** Quantitative image analysis produces a data matrix that can be aggregated and disaggregated to varying degrees in both spatial (A) and temporal (B) dimensions, creating distinct but inter-operable data hierarchies. (A) Statistical measures of Cell Populations represent the most aggregated state of data in the Spatial Data Hierarchy. Disaggregation of data to Single Cells provides the Cellular Resolution Level. Each cell contains a CMAC Population; therefore Aggregated CMAC Population Statistics are necessary to describe the adhesion cohort of an individual cell. In combination with descriptions of Individual Cell Properties, these two spatial scales of data, collectively termed Single Cell Scale, are used almost exclusively in this study (see green outline, A). Features describing these scales are described in Supporting Table S1. The properties of Single CMACs are also independently recorded, providing access to Individual CMAC Properties as the maximal level of spatial data disaggregation (data from this spatial scale is presented in Supporting Figure S2, C and D, based on features defined in Supporting Table S2). (B) Image data is acquired over 8 h and therefore the combination of data from all time-points represents the maximal level of data aggregation in the Temporal Data Hierarchy. Varying degrees of data disaggregation via Arbitrary Time Sampling, as employed in Supporting Figure S2, can provide indications regarding the stability of experimental conditions over time. Because migrating cells can enter and exit imaging fields at any time, per-cell data may be aggregated to reflect individual Cell Observation Periods. Within each cell, data defining the properties of each CMAC may be aggregated over the lifetime of each CMAC. Alternatively, as with Single Cell and Cell Population data, CMAC data may be disaggregated to reflect Instantaneous Dynamics defined at the maximal image sampling frequency (5 min). Instantaneous Dynamics data is used in most cases throughout this study.
(EPS)

Figure S2 **Multivariate quantitative analyses indicate high inter- and intra-experimental data consistency.** PCA analysis based on 88 Cell-Level variables (A, 2446 data points = individual cells at single time-points, analyzed variables defined in Supporting Table S1) or 29 CMAC-Level variables (C, 71076 data points = individual CMACs at single time-points, analyzed variables defined in Supporting Table S2) color-coded by experimental repeat date reveal high overlap between data derived during independent experiments. Similar analyses of Cell-Level (B) or CMAC-Level (D) data color-coded by intra-experimental time (four non-overlapping 2 h windows) also show excellent consistency indicating a stable-steady state during experimentation.
(TIFF)

Figure S3 **Recorded organizational and behavioral features are quantitatively linked.** (A) Principal component analysis (PCA) was performed for all control cell observations based on all 87 *organizational* features. An expectation maximization (EM)-algorithm for Gaussian mixture models using principal components 1–20 (including >99% of total variance) was employed to assign control cell data into two subpopulations. This was repeated ten times to achieve an optimized assignment as determined by assessment of relative inter- and intra-group variability using the Akaike information criterion (AIC). This procedure was replicated for the assignment of control cell data into between 2 and 8 subpopulations, revealing that control cell *organizational* feature data is optimally represented as four subpopulations. Multivariate analysis of variance (MANOVA) allowed rejection of hypotheses that 1, 2 or 3 sub-populations exist (P values = 0), indicating support for the existence of 4 or more subpopulations. Accordingly, control cell observations were assigned to one of four subpopulations (G1–G4), with assignments finalized based on the lowest achieved AIC value following 100 randomly-seeded EM-algorithm iterations. Given these assignments, canonical vectors analysis (CVA) was used to visualize the multivariate distributions of subpopulations G1–G4 based on the original 87 *organizational* features. Subpopulations G1 (blue) and G3 (orange) partially overlap, with G4 (red) relatively proximal and G2 (green) relatively distal. Both standardized Mahalanobis distance measurement (B, blue - near; red - far) and independent hierarchical clustering (C) confirm the structure of the *organizational* feature-based difference hierarchy for these control cell subpopulations. (D) To compare this *organizational* difference hierarchy with the corresponding *behavioral* difference hierarchy, we visualized the probability distribution function (P.D.F.) for *Instantaneous Cell Speed (ICS, behavioral* feature*)* associated with each control cell subpopulation defined in A. In correspondence to results in A–C, G1 and G3 subpopulation *behavior* was highly analogous (KS-test p-value G1 vs G3 = 0.56) with a bias towards fast moving cells, G4 had a bias towards moderately motile cells (KS-test p-value G1 vs G4 = 0), and G2 was biased towards slow moving cells (KS-test p-value G1 vs G2 = 0). Thus, *organizational* and *behavioral* difference hierarchies were ordinally equivalent when control cell subpopulations were defined based on *organizational* state. (E) To complement this methodology, *behavioral* stratification of control cells was performed according to quintiles (**S1** [1–20%, dark blue], **S2** [21–40%, light blue], **S3** [41–60%, green], **S4** [61–80%, orange], **S5** [81–100%, red]) of the *ICS* distribution. (F) CVA based on all 87 *organizational* variables (excluding *ICS*) revealed the progressive divergence of these subpopulations in accordance with differences in *behavior*. Standardized Mahalanobis distance measurement (G) and independent hierarchical clustering (H) confirmed the ordinal equivalence between the difference hierarchies defined by *organizational* and *behavioral* features when control cell subpopulations were assigned based on *behavioral* state.
(EPS)

Figure S4 **Assessing the efficacy and impact of ROCK and Rho signaling perturbations.** (A) CVA analysis of control (DMSO, blue), ROCK-inhibited (ROCK, red) and Rho-activated (Rho, yellow) cells defined by all Cell-Level variables reveals near complete segregation of these populations as well as high consistency within these experimental conditions. MANOVA allows rejection of the hypotheses that there are one or two cell populations (P values = 0), thereby supporting the existence of at least 3 independent cell populations corresponding to treatment conditions. These results support the general efficacy of these perturbations. (B) KS-tests comparing the distributions of each variable (compact feature names defined in Supporting Table S1) between control and either Rho-activated (Rho, left column) or ROCK-inhibited (ROCK, right column) conditions reveal a broad array of perturbation effects (white circles denote KS-test P-values <0.05, colors indicate aggregate inter-distribution distances (blue = proximal, red = distal)). (C–H) Selected probability distribution functions (DMSO (control) = blue, ROCK (ROCK-inhibited) = red, Rho (Rho-activated) = yellow) are highlighted here to provide comparison to previously published analyses of Rho and ROCK manipulation, thereby confirming the expected impacts of these perturbations. Specifically, Rho activation significantly reduced cell migration speeds compared to control [56]–[58], while ROCK inhibition increased the frequency and maximal speeds of fast moving cells (C) [59]–[61]. Rho activation promoted rounder, more compact cell morphologies indicative of increased contractility (D) [62], [63], while ROCK inhibition lead to a more protrusive phenotype [64], [65]. Both Rho activation and ROCK inhibition promoted an increase in CMAC number per cell, though ROCK inhibition had a markedly stronger effect (E) [66], [67]. ROCK inhibition dramatically reduced the median area of CMACs (F) [60], [63], [68], while Rho activation had inverse effects [67], [69], [70]. Correspondingly, ROCK inhibition accelerated the turnover of CMACs, while Rho lead to a significant increase in mean CMAC lifetime per cell (G) [61], [71]–[73]. Analysis of intra-CMAC colocalization between EGFP-Paxillin and RubyRed-LifeAct showed that co-recruitment of these components within CMACs was enhanced by Rho activation and inhibited by ROCK inhibition (H).
(EPS)

Table S1 **Definition of Organizational and Behavioral features.** This table includes classification and description of those features defining cell migration system *organization* and *behavior* that are discussed herein. Variables are alternatively classified according to several criteria: relevance to *organization* or *behavior*; Variable Class (Cell Dynamics, Cell Morphology, CMAC Dynamics, CMAC Intensity, CMAC Localization, CMAC Morphology, Colocalization); Spatial Scaling (Cellular, CMAC population per Cell); and Temporal Scaling (Instantaneous, CMAC Lifetime). Full variable names are listed, as well as a shortened name code (used due to space restrictions in Figures 3 and 4 and Supporting Figure S3). The corresponding units and a concise description are also included. Additional terms are defined above relevant columns.
(XLSX)

Table S2 **Definition of CMAC variables.** This table includes classification and description of those variables defining instantaneous CMAC features (considered only in Supporting Figure S2 C and D). Variables are alternatively classified according to several criteria: Variable Class (CMAC Morphology, CMAC Dynamics, CMAC Intensity, CMAC Localization, CMAC Colocalization); Spatial Scaling (Individual CMAC values only); and Temporal Scaling (Instantaneous values only). Full variable names are listed, along with the corresponding units and a concise variable description. Additional terms are defined above relevant columns.
(XLSX)

Movie S1 **Control cell migration.** Control (DMSO-treated) H1299-P/L cells expressing EGFP-Paxillin (green, left panel) and RubyRed-LifeAct (red, left panel) were imaged at 5 min intervals for 8 h. EGFP-Paxillin is concentrated within punctate cell-matrix adhesion complexes (CMACs) while RubyRed-LifeAct associates with the F-Actin cytoskeleton. After raw confocal image acquisition (left panel), automated image analysis facilitated segmentation of individual cells based on RubyRed-LifeAct fluorescence (cell boundary indicated by dark blue outline, overlaying EGFP-Paxillin channel, center panel). Subsequently, CMACs within each cell were segmented (CMACs detected based on EGFP-Paxillin fluorescence, indicated by red outlines, center panel). Finally, the centroids of both cells and their constituent CMACs were tracked via nearest neighbor analysis to facilitate time-resolved analyses of dynamic migratory processes (right panel). CMAC trajectories are color-coded for time, ≤10 time points shown. Scale bars in 1^st^ frame = 50 µm.
(MOV)

Movie S2 **Cell migration following ROCK-inhibition.** ROCK-inhibitor-treated H1299-P/L cells expressing EGFP-Paxillin (green, left panel) and RubyRed-LifeAct (red, left panel) were imaged at 5 min intervals for 8 h. Note the reduced size and stability of EGFP-Paxillin-labeled CMACs, and the increased mobility and protrusiveness of this representative cell. After raw confocal image acquisition (left panel), automated image analysis facilitated segmentation of individual cells based on RubyRed-LifeAct fluorescence (cell boundary indicated by dark blue outline, overlaying EGFP-Paxillin channel, center panel). Subsequently, CMACs within each cell were segmented (CMACs detected based on EGFP-Paxillin fluorescence, indicated by red outlines, center panel). Finally, the centroids of both cells and their constituent CMACs were tracked via nearest neighbor analysis to facilitate time-resolved analyses of dynamic migratory processes (right panel). CMAC trajectories are color-coded for time, ≤10 time points shown. Scale bars in 1^st^ frame = 50 µm.
(MOV)

Movie S3 **Cell migration following Rho-activation.** Rho-activator-treated H1299-P/L cells expressing EGFP-Paxillin (green, left panel) and RubyRed-LifeAct (red, left panel) were imaged at 5 min intervals for 8 h. Note the increased size and stability of EGFP-Paxillin-labeled CMACs, as well as the reduced area and motility of this representative cell. After raw confocal image acquisition (left panel), automated image analysis facilitated segmentation of individual cells based on RubyRed-LifeAct fluorescence (cell boundary indicated by dark blue outline, overlaying EGFP-Paxillin channel, center panel). Subsequently, CMACs within each cell were segmented (CMACs detected based on EGFP-Paxillin fluorescence, indicated by red outlines, center panel). Finally, the centroids of both cells and their constituent CMACs were tracked via nearest neighbor analysis to facilitate time-resolved analyses of dynamic migratory processes (right panel). CMAC trajectories are color-coded for time, ≤10 time points shown. Scale bars in 1^st^ frame = 50 µm.
(MOV)

## References

[1] Horwitz R, Webb D (2003) Cell migration. Curr Biol 13: R756–R759. 14521851 10.1016/j.cub.2003.09.014

[2] Friedl P, Wolf K (2010) Plasticity of cell migration: a multiscale tuning model. J Cell Biol 188: 11–19. 19951899 10.1083/jcb.200909003 PMC2812848

[3] Humphries JD, Byron A, Bass MD, Craig SE, Pinney JW, et al (2009) Proteomic analysis of integrin-associated complexes identifies RCC2 as a dual regulator of Rac1 and Arf6. Sci STKE 2: ra51. 10.1126/scisignal.2000396 PMC2857963 19738201

[4] Schiller HB, Friedel CC, Boulegue C, Fässler R (2011) Quantitative proteomics of the integrin adhesome show a myosin II-dependent recruitment of LIM domain proteins. EMBO Rep 12: 259–266. 21311561 10.1038/embor.2011.5 PMC3059911

[5] Kuo JC, Han X, Hsiao CT, Yates IiiJR, Waterman CM (2011) Analysis of the myosin-II-responsive focal adhesion proteome reveals a role for [beta]-Pix in negative regulation of focal adhesion maturation. Nat Cell Biol 13: 383–393. 21423176 10.1038/ncb2216 PMC3279191

[6] Byron A, Humphries JD, Bass MD, Knight D, Humphries MJ (2011) Proteomic analysis of integrin adhesion complexes. Sci Signal 4: pt2. 21467297 10.1126/scisignal.2001827

[7] Geiger T, Zaidel-Bar R (2012) Opening the floodgates: proteomics and the integrin adhesome. Curr Opin Cell Biol 24: 562–568. 22728062 10.1016/j.ceb.2012.05.004

[8] Lock JG, Wehrle-Haller B, Stromblad S (2008) Cell-matrix adhesion complexes: master control machinery of cell migration. Semin Cancer Biol 18: 65–76. 18023204 10.1016/j.semcancer.2007.10.001

[9] Oltvai ZN, Barabasi AL (2002) Systems biology. Life’s complexity pyramid. Science 298: 763–764. 12399572 10.1126/science.1078563

[10] Ravasz E, Somera AL, Mongru DA, Oltvai ZN, Barabasi AL (2002) Hierarchical organization of modularity in metabolic networks. Science 297: 1551–1555. 12202830 10.1126/science.1073374

[11] Welf ES, Haugh JM (2011) Signaling pathways that control cell migration: models and analysis. Wiley Interdiscip Rev Syst Biol Med 3: 231–240. 21305705 10.1002/wsbm.110 PMC3052860

[12] Karsenti E (2008) Self-organization in cell biology: a brief history. Nat Rev Mol Cell Biol 9: 255–262. 18292780 10.1038/nrm2357

[13] Strohman RC (1997) The coming Kuhnian revolution in biology. Nat Biotechnol 15: 194–200. 9062910 10.1038/nbt0397-194

[14] Lazebnik Y (2002) Can a biologist fix a radio?–Or, what I learned while studying apoptosis. Cancer Cell 2(3): 179–182. 12242150 10.1016/s1535-6108(02)00133-2

[15] Vilela M, Danuser G (2011) What’s wrong with correlative experiments? Nat Cell Biol 13: 1011. 21892139 10.1038/ncb2325

[16] Kim DH, Wirtz D (2012) Focal adhesion size uniquely predicts cell migration. FASEB J 27: 1351–1361. 23254340 10.1096/fj.12-220160 PMC3606534

[17] Ku C-J, Wang Y, Weiner OD, Altschuler SJ, Wu LF (2012) Network Crosstalk Dynamically Changes during Neutrophil Polarization. Cell 149: 1073–1083. 22632971 10.1016/j.cell.2012.03.044 PMC3614011

[18] Geiger B, Yamada KM (2011) Molecular architecture and function of matrix adhesions. Cold Spring Harb Perspect Biol 3: a005033. 21441590 10.1101/cshperspect.a005033 PMC3101841

[19] Parsons JT, Horwitz AR, Schwartz MA (2010) Cell adhesion: integrating cytoskeletal dynamics and cellular tension. Nat Rev Mol Cell Biol 11: 633–643. 20729930 10.1038/nrm2957 PMC2992881

[20] Gardel ML, Schneider IC, Aratyn-Schaus Y, Waterman CM (2010) Mechanical integration of actin and adhesion dynamics in cell migration. Annu Rev Cell Dev Biol 26: 315–333. 19575647 10.1146/annurev.cellbio.011209.122036 PMC4437624

[21] Lock JG, Stromblad S (2010) Systems microscopy: an emerging strategy for the life sciences. Exp Cell Res 316: 1438–1444. 20381488 10.1016/j.yexcr.2010.04.001

[22] Sachs K, Perez O, Pe’er D, Lauffenburger DA, Nolan GP (2005) Causal protein-signaling networks derived from multiparameter single-cell data. Science 308: 523–529. 15845847 10.1126/science.1105809

[23] Janes KA, Gaudet S, Albeck JG, Nielsen UB, Lauffenburger DA, et al (2006) The response of human epithelial cells to TNF involves an inducible autocrine cascade. Cell 124: 1225–1239. 16564013 10.1016/j.cell.2006.01.041

[24] Tkachenko E, Sabouri-Ghomi M, Pertz O, Kim C, Gutierrez E, et al (2011) Protein kinase A governs a RhoA-RhoGDI protrusion-retraction pacemaker in migrating cells. Nat Cell Biol 13: 660–667. 21572420 10.1038/ncb2231 PMC3746034

[25] Natarajan M, Lin KM, Hsueh RC, Sternweis PC, Ranganathan R (2006) A global analysis of cross-talk in a mammalian cellular signalling network. Nat Cell Biol 8: 571–580. 16699502 10.1038/ncb1418

[26] Granger CWJ (1969) Investigating causal relations by econometric models and cross-spectral methods. Econometrica: Journal of the Econometric Society 424–438.

[27] Wiener N (1956) The theory of prediction. Modern mathematics for engineers. 165–190.

[28] Bressler SL, Seth AK (2011) Wiener–Granger causality: a well established methodology. Neuroimage 58: 323–329. 20202481 10.1016/j.neuroimage.2010.02.059

[29] Gupton SL, Waterman-Storer CM (2006) Spatiotemporal feedback between actomyosin and focal-adhesion systems optimizes rapid cell migration. Cell 125: 1361–1374. 16814721 10.1016/j.cell.2006.05.029

[30] Hou Y, Hedberg S, Schneider IC (2012) Differences in adhesion and protrusion properties correlate with differences in migration speed under EGF stimulation. BMC Biophys 5: 8. 22577847 10.1186/2046-1682-5-8 PMC3414788

[31] Webb DJ, Donais K, Whitmore LA, Thomas SM, Turner CE, et al (2004) FAK–Src signalling through paxillin, ERK and MLCK regulates adhesion disassembly. Nat Cell Biol 6: 154–161. 14743221 10.1038/ncb1094

[32] Winograd-Katz SE, Itzkovitz S, Kam Z, Geiger B (2009) Multiparametric analysis of focal adhesion formation by RNAi-mediated gene knockdown. J Cell Biol 186: 423–436. 19667130 10.1083/jcb.200901105 PMC2728402

[33] Li Z, Lock JG, Olofsson H, Kowalewski JM, Teller S, et al (2010) Integrin-mediated Cell Attachment Induces a PAK4-dependent Feedback Loop Regulating Cell Adhesion through Modified Integrin αvβ5 Clustering and Turnover. Mol Biol Cell 21: 3317–3329. 20719960 10.1091/mbc.E10-03-0245 PMC2947468

[34] Pe’er D (2005) Bayesian network analysis of signaling networks: a primer. Science Signaling 2005: pl4. 10.1126/stke.2812005pl4 15855409

[35] Huang S, Ingber DE (2000) Shape-dependent control of cell growth, differentiation, and apoptosis: switching between attractors in cell regulatory networks. Exp Cell Res 261: 91–103. 11082279 10.1006/excr.2000.5044

[36] Maree AF, Grieneisen VA, Edelstein-Keshet L (2012) How cells integrate complex stimuli: the effect of feedback from phosphoinositides and cell shape on cell polarization and motility. PLoS Comput Biol 8: e1002402. 22396633 10.1371/journal.pcbi.1002402 PMC3291540

[37] Jafari-Mamaghani M (2013) Non-parametric Analysis of Granger Causality Using Local Measures of Divergence. Applied Mathematical Sciences 7: 4107–4136.

[38] Bar-Yam Y (2004) A mathematical theory of strong emergence using multiscale variety. Complexity 9: 15–24.

[39] Welf ES, Naik UP, Ogunnaike BA (2011) Probabilistic modeling and analysis of the effects of extra-cellular matrix density on the sizes, shapes, and locations of integrin clusters in adherent cells. BMC Biophys 4: 15. 21827670 10.1186/2046-1682-4-15 PMC3179437

[40] Coló GP, Hernández-Varas P, Lock J, Bartolome RA, Arellano-Sanchez N, et al (2012) Focal adhesion disassembly is regulated by a RIAM to MEK-1 pathway. J Cell Sci 125: 5338–5352. 22946047 10.1242/jcs.105270

[41] Ezratty EJ, Bertaux C, Marcantonio EE, Gundersen GG (2009) Clathrin mediates integrin endocytosis for focal adhesion disassembly in migrating cells. J Cell Biol 187: 733–747. 19951918 10.1083/jcb.200904054 PMC2806590

[42] Mogilner A, Keren K (2009) The shape of motile cells. Curr Biol 19: R762–R771. 19906578 10.1016/j.cub.2009.06.053 PMC2864320

[43] Barnhart EL, Lee KC, Keren K, Mogilner A, Theriot JA (2011) An adhesion-dependent switch between mechanisms that determine motile cell shape. PLoS Biol 9: e1001059. 21559321 10.1371/journal.pbio.1001059 PMC3086868

[44] Yam PT, Wilson CA, Ji L, Hebert B, Barnhart EL, et al (2007) Actin-myosin network reorganization breaks symmetry at the cell rear to spontaneously initiate polarized cell motility. J Cell Biol 178: 1207–1221. 17893245 10.1083/jcb.200706012 PMC2064654

[45] Sahai E, Garcia-Medina R, Pouyssegur J, Vial E (2007) Smurf1 regulates tumor cell plasticity and motility through degradation of RhoA leading to localized inhibition of contractility. J Cell Biol 176: 35–42. 17190792 10.1083/jcb.200605135 PMC2063621

[46] Wolfenson H, Bershadsky A, Henis YI, Geiger B (2011) Actomyosin-generated tension controls the molecular kinetics of focal adhesions. J Cell Sci 124: 1425–1432. 21486952 10.1242/jcs.077388 PMC3078811

[47] Sawada Y, Sheetz MP (2002) Force transduction by Triton cytoskeletons. J Cell Biol 156: 609–615. 11839769 10.1083/jcb.200110068 PMC2174068

[48] Zamir E, Geiger B, Kam Z (2008) Quantitative multicolor compositional imaging resolves molecular domains in cell-matrix adhesions. PLoS One 3: e1901. 18382676 10.1371/journal.pone.0001901 PMC2270910

[49] Riveline D, Zamir E, Balaban NQ, Schwarz US, Ishizaki T, et al (2001) Focal contacts as mechanosensors: externally applied local mechanical force induces growth of focal contacts by an mDia1-dependent and ROCK-independent mechanism. J Cell Biol 153: 1175–1186. 11402062 10.1083/jcb.153.6.1175 PMC2192034

[50] Smilenov L, Forsberg E, Zeligman I, Sparrman M, Johansson S (1992) Separation of fibronectin from a plasma gelatinase using immobilized metal affinity chromatography. FEBS Lett 302: 227–230. 1318226 10.1016/0014-5793(92)80447-o

[51] Box GEP, Cox DR (1964) An analysis of transformations. J R Stat Soc Series B Stat Methodol 26: 211–252.

[52] Dempster AP, Laird NM, Rubin DB (1977) Maximum likelihood from incomplete data via the EM algorithm. J R Stat Soc Series B Stat Methodol 39: 1–38.

[53] Akaike H (1974) A new look at the statistical model identification. Automatic Control, IEEE Transactions on 19: 716–723.

[54] Rao CR (1948) The utilization of multiple measurements in problems of biological classification. J R Stat Soc Series B Stat Methodol 10: 159–203.

[55] Zou H, Hastie T (2005) Regularization and variable selection via the elastic net. J R Stat Soc Series B Stat Methodol 67: 301–320.

[56] Nobes CD, Hall A (1999) Rho GTPases control polarity, protrusion, and adhesion during cell movement. J Cell Biol 144: 1235–1244. 10087266 10.1083/jcb.144.6.1235 PMC2150589

[57] Ridley AJ, Comoglio PM, Hall A (1995) Regulation of scatter factor/hepatocyte growth factor responses by Ras, Rac, and Rho in MDCK cells. Mol Cell Biol 15: 1110–1122. 7823927 10.1128/mcb.15.2.1110 PMC232019

[58] Takaishi K, Sasaki T, Kato M, Yamochi W, Kuroda S, et al (1994) Involvement of Rho p21 small GTP-binding protein and its regulator in the HGF-induced cell motility. Oncogene 9: 273–279. 8302589

[59] Totsukawa G, Wu Y, Sasaki Y, Hartshorne DJ, Yamakita Y, et al (2004) Distinct roles of MLCK and ROCK in the regulation of membrane protrusions and focal adhesion dynamics during cell migration of fibroblasts. J Cell Biol 164: 427–439. 14757754 10.1083/jcb.200306172 PMC2172229

[60] Pasapera AM, Schneider IC, Rericha E, Schlaepfer DD, Waterman CM (2010) Myosin II activity regulates vinculin recruitment to focal adhesions through FAK-mediated paxillin phosphorylation. J Cell Biol 188: 877–890. 20308429 10.1083/jcb.200906012 PMC2845065

[61] Ridley AJ (2001) Rho GTPases and cell migration. J Cell Sci 114: 2713–2722. 11683406 10.1242/jcs.114.15.2713

[62] Tkach V, Bock E, Berezin V (2005) The role of RhoA in the regulation of cell morphology and motility. Cell Motil Cytoskeleton 61: 21–33. 15776463 10.1002/cm.20062

[63] Riento K, Ridley AJ (2003) Rocks: multifunctional kinases in cell behaviour. Nat Rev Mol Cell Biol 4: 446–456. 12778124 10.1038/nrm1128

[64] Tsuji T, Ishizaki T, Okamoto M, Higashida C, Kimura K, et al (2002) ROCK and mDia1 antagonize in Rho-dependent Rac activation in Swiss 3T3 fibroblasts. J Cell Biol 157: 819–830. 12021256 10.1083/jcb.200112107 PMC2173402

[65] Salhia B, Rutten F, Nakada M, Beaudry C, Berens M, et al (2005) Inhibition of Rho-kinase affects astrocytoma morphology, motility, and invasion through activation of Rac1. Cancer Res 65: 8792. 16204049 10.1158/0008-5472.CAN-05-0160

[66] Ridley AJ, Hall A (1992) The small GTP-binding protein rho regulates the assembly of focal adhesions and actin stress fibers in response to growth factors. Cell 70: 389–399. 1643657 10.1016/0092-8674(92)90163-7

[67] Chrzanowska-Wodnicka M, Burridge K (1996) Rho-stimulated contractility drives the formation of stress fibers and focal adhesions. J Cell Biol 133: 1403–1415. 8682874 10.1083/jcb.133.6.1403 PMC2120895

[68] Struckhoff AP, Vitko JR, Rana MK, Davis CT, Foderingham KE, et al (2010) Dynamic regulation of ROCK in tumor cells controls CXCR4-driven adhesion events. J Cell Sci 123: 401–412. 20053635 10.1242/jcs.052167 PMC2816185

[69] Amano M, Chihara K, Kimura K, Fukata Y, Nakamura N, et al (1997) Formation of actin stress fibers and focal adhesions enhanced by Rho-kinase. Science 275: 1308–1311. 9036856 10.1126/science.275.5304.1308

[70] Ishizaki T, Naito M, Fujisawa K, Maekawa M, Watanabe N, et al (1997) p160ROCK, a Rho-associated coiled-coil forming protein kinase, works downstream of Rho and induces focal adhesions. FEBS Lett 404: 118–124. 9119047 10.1016/s0014-5793(97)00107-5

[71] Schober M, Raghavan S, Nikolova M, Polak L, Pasolli HA, et al (2007) Focal adhesion kinase modulates tension signaling to control actin and focal adhesion dynamics. J Cell Biol 176: 667–680. 17325207 10.1083/jcb.200608010 PMC2064024

[72] Yamana N, Arakawa Y, Nishino T, Kurokawa K, Tanji M, et al (2006) The Rho-mDia1 pathway regulates cell polarity and focal adhesion turnover in migrating cells through mobilizing Apc and c-Src. Mol Cell Biol 26: 6844–6858. 16943426 10.1128/MCB.00283-06 PMC1592856

[73] Webb DJ, Parsons JT, Horwitz AF (2002) Adhesion assembly, disassembly and turnover in migrating cells–over and over and over again. Nat Cell Biol 4: E97–E100. 11944043 10.1038/ncb0402-e97

