# Supplementary figures and images for: Plasticity in the Macromolecular-Scale Causal Networks of Cell Migration
Source: PLoS One. 2014 Feb 28;9(2):e90593. doi: 10.1371/journal.pone.0090593 (PMC3938764; doi:10.1371/journal.pone.0090593)

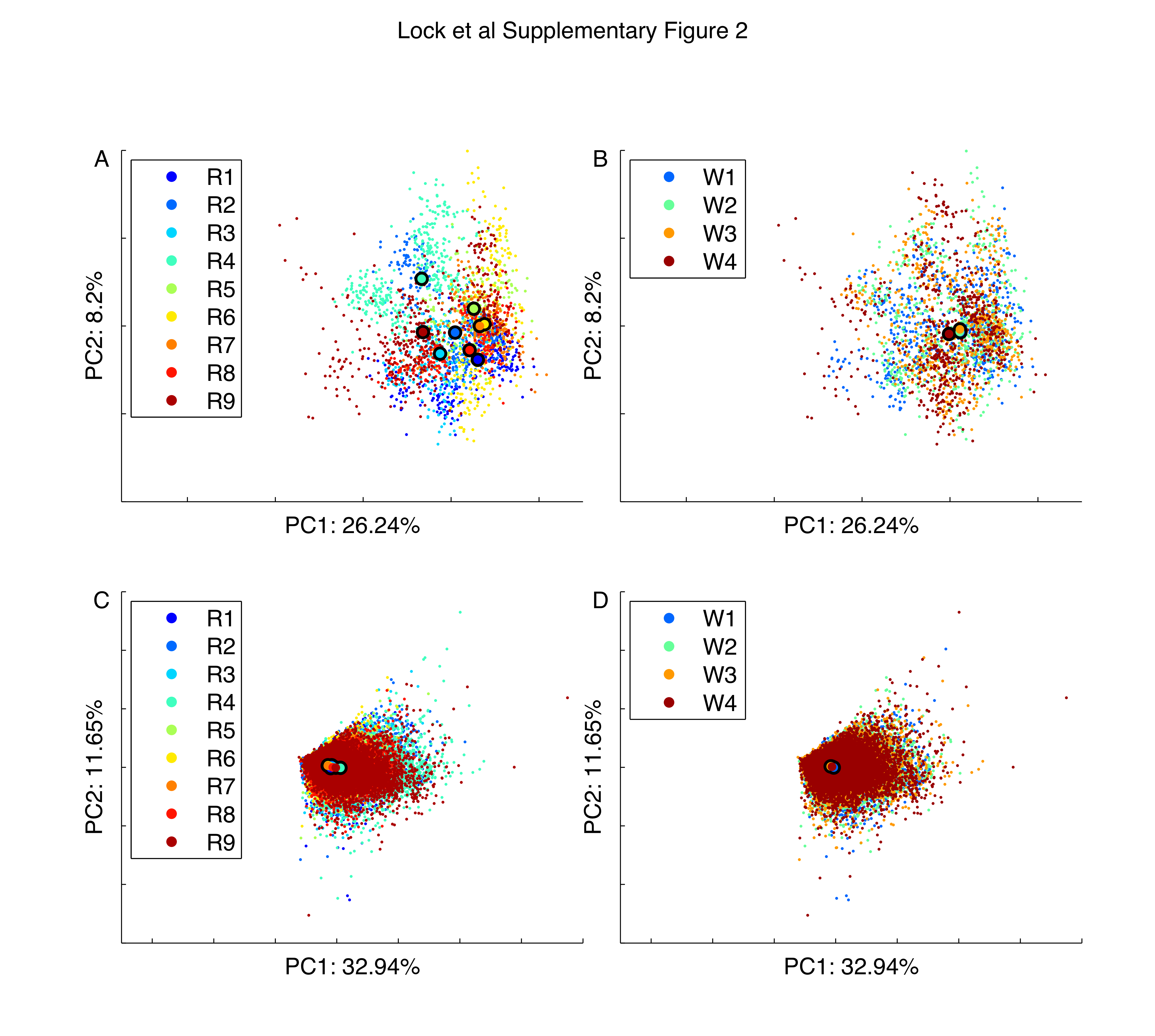

Supplement: Figure S2 — Multivariate quantitative analyses indicate high inter- and intra-experimental data consistency. PCA analysis based on 88 Cell-Level variables (A, 2446 data points = individual cells at single time-points, analyzed variables defined in Supporting Table S1) or 29 CMAC-Level variables (C, 71076 data points = individual CMACs at single time-points, analyzed variables defined in Supporting Table S2) color-coded by experimental repeat date reveal high overlap between data derived during independent experiments. Similar analyses of Cell-Level (B) or CMAC-Level (D) data color-coded by intra-experimental time (four non-overlapping 2 h windows) also show excellent consistency indicating a stable-steady state during experimentation. (TIFF) [file pone.0090593.s002.tif]
